# β-carotene enhances drought tolerance in fenugreek by modulating antioxidant defense and redox homeostasis

**DOI:** 10.1080/15592324.2026.2716518

**Published:** 2026-09-17

**Authors:** Minahal Hussain, Tahrim Ramzan, Muhammad Shahbaz, Arslan Haider, Muhammad Ahmad, Ejaz Ahmad Waraich, Usman Zulfiqar, Hossam S. El-Beltagi, Abdulrahman Alasmari, Mohammed S. Alotaibi, Mayank Anand Gururani

**Affiliations:** a Department of Botany, University of Agriculture, Faisalabad, Pakistan; b College of Grassland and Pasture, Northwest Agriculture and Forestry University, Yangling, Shaanxi, China; c West Central Research Extension and Education Center, University of Nebraska-Lincoln, NE, USA; d Department of Agronomy & Horticulture, University of Nebraska-Lincoln, Lincoln, NE, USA; e Department of Agronomy, University of Agriculture, Faisalabad, Pakistan; f Department of Agronomy, Faculty of Agriculture and Environment, The Islamia University of Bahawalpur, Bahawalpur, Pakistan; g Department of Biology, Nakhchivan State University, Nakhchivan, Azerbaijan; h Agricultural Biotechnology Department, College of Agriculture and Food Sciences, King Faisal University, Al-Ahsa, Saudi Arabia; i Department of Biology, Faculty of Science, University of Tabuk, Tabuk, Saudi Arabia; j Biodiversity Genomics Unit, Faculty of Science, University of Tabuk, Tabuk, Saudi Arabia; k Department of Biology, Turabah University College, Taif University, Taif, Saudi Arabia; l Department of Biology, College of Science, United Arab Emirates University, Al Ain, United Arab Emirates

**Keywords:** Antioxidants, β-carotene, drought, fenugreek, ionic attributes, osmolytes

## Abstract

Drought stress is one of the main abiotic factors that modulates the morphology and physiology of crops. This study investigated the effect of foliar application of *β*-carotene on the growth, physiological, and biochemical responses of fenugreek (*Trigonella foenum-graecum* L.) under drought stress conditions. A pot experiment was conducted using two varieties, Kasuri and Local, under two drought stress levels (control and 50% field capacity), and three *β*-carotene concentrations (0, 100, and 200 ppm) were applied. Drought stress significantly declined shoot fresh weight up to 35.02% and 58.04%, and shoot length to 17.12% and 17.14%, while increasing the root fresh weight by 133% and 26.2% and the root length to 109.1% and 13.4%, respectively, in the Kasuri methi and Local. Drought stress decreases the total Chl. by 55.4% and 59.3% and carotenoids 42.1% and 59.3% and increased the MDA by 6.35% and 24.2%, respectively, and the content of hydrogen peroxides increased by 12.05% and 44.2% in Kasuri and Local as compared to control. By the application of 200  ppm *β*-carotene, the shoot fresh weight increased by 95.06% and 66.7%, the shoot length increased by 49.6% and 44.5%, and the total Chl. increased by 194.3% and 144.3%, and carotenoids 71.6% and 63%, and MDA decreased by 14.7% and 15.8%, hydrogen peroxides 26.6% and 27.8%, in Kasuri methi and Local under drought stress conditions. Additionally, with the application of *β*-carotene, antioxidant enzyme activities (SOD, POD, and CAT) and osmoprotectants (total soluble proteins and sugars) improved significantly, indicating enhanced oxidative defense. Overall, foliar *β*-carotene application, especially at 200 ppm, proved highly effective in improving fenugreek’s drought tolerance by enhancing antioxidant capacity, maintaining pigment stability, and supporting metabolic homeostasis, thereby highlighting its potential role in sustainable crop management under water-limited conditions.

## 1. Introduction

Fenugreek (*Trigonella foenum-graecum* L.) is an annual, self-pollinated legume crop belonging to the Fabaceae family, with a diploid chromosome number of 2n = 16. The Latin name for the genus Trigonella means “little triangle,” relating to the trait of triangular-shaped flowers, while the species name *foenum-graecum* translates to “Greek hay,” reflecting its early use as forage.^1^ ^,^ ^2^ Fenugreek is cultivated globally across diverse agro-ecological zones due to its multipurpose nature used as a spice, vegetable, medicinal herb and fodder it improves soil fertility through its nitrogen-fixing ability, contributing to sustainable agriculture by reducing the need for synthetic fertilizers.^3^ Its seeds are rich in proteins, dietary fibers, essential fatty acids (oleic, linoleic, linolenic), alkaloids (trigonelline), and steroidal saponins (diosgenin), which collectively contribute to its therapeutic potential, including antidiabetic, anti-inflammatory, antioxidant, hypocholesterolemic, and galactagogue effects.^4^ The seeds and leaves of fenugreek are valued for their rich nutrient content and therapeutic bioactive compounds, which include flavonoids and carotenoids.^5^ Fenugreek leaves are particularly rich in essential nutrients, such as vitamin C, calcium, iron, as well as provitamin, a compound like *β*-carotene. The presence of *β*-carotene in the leaves contributes to their antioxidant potential and nutritional significance, making fenugreek an important leafy vegetable in diets across South Asia and North Africa.^6^

Environmental stressors can significantly influence plant growth, often causing substantial damage to biomass production when stress becomes severe.^7–10^ These stress factors such as salinity, drought or temperature extremes may develop either gradually or abruptly.^11–13^ Prolonged insufficient water supply is a hallmark of drought stress.^14^ ^,^ ^15^ By 2025, Pakistan's average water deficit was 31%. Compared to other provinces in Pakistan, Baluchistan and Sindh are more vulnerable to drought.^16^ ^,^ ^17^ Additionally, drought stress impairs important plant metabolic processes, including photophosphorylation, electron transport, and protein synthesis, which eventually stunts growth and development.^18–20^

Drought stress is one of the most critical abiotic stressors limiting plant growth, development, and crop yield worldwide.^16^ ^,^ ^21^ During drought conditions, the shortage of water lowers a plant's relative water content and leaf water potential, adversely affecting key metabolic activities, transpiration, ion uptake, pressure of turgor, and cell expansion.^22^ Additionally, drought-induced lower respiration, nutrient translocation, regulation of phytohormones, as well as stomatal closure, limit the uptake of carbon dioxide, which reduces chlorophyll content and significantly hampers photosynthetic efficiency.^16^ ^,^ ^23^ Moreover, drought conditions raise the generation of reactive oxygen species that can oxidatively harm essential cellular constituents such as proteins, DNA, and lipids.^24^ ^,^ ^25^ This oxidative stress can harm cellular function if it is not controlled by the plant's defence mechanisms and may ultimately lead to plant death.^14^ ^,^ ^26^ However, exogenous application of plant growth regulators can improve the resilience to abiotic stresses.^27^

Carotenoids are powerful antioxidants that regulate photosynthesis and promote plant growth. They help protect plant tissues by eliminating free radicals and preventing oxidative damage, which are also closely involved in how plants respond to drought, high light intensity and severe temperatures.^28^ Among these pigments, *β*-carotene stands out as an essential component for both plant function and human health. *β*-carotene is found naturally in a variety of fruits and vegetables, including spinach, sweet potatoes, and carrots, and is responsible for their characteristic reddish-orange color.^29^ In humans, *β*-carotene serves as a precursor to vitamin A (provitamin A), which the body converts into retinol, an active form of the vitamin. This conversion supports crucial physiological functions, such as preserving good vision, bolstering the immune system and encouraging healthy skin.^30^

*β*-carotene, a powerful antioxidant, plays an important role against oxidative stress and reduces the risk of chronic diseases such as cardiovascular disease and certain cancers.^31^ ^,^ ^32^ In plants, *β*-carotene plays a crucial role in photosynthesis by aiding in light absorption and protecting chlorophyll molecules from oxidative damage.^33^ Beyond its physiological function in plants, *β*-carotene is widely recognized for its scavenging free radicals and preventing oxidative damage, which is also closely involved in how plants respond to drought, high light intensity, and severe temperatures.^28^

Despite the known presence of *β*-carotene in fenugreek, there is limited research has focused exclusively on its physiological or molecular effects. Most existing studies assess total antioxidant activity or vitamin A equivalence but do not isolate *β*-carotene's role in fenugreek bio efficacy. Addressing this gap is essential for optimizing the use of fenugreek in functional food development.^34^ This study hypothesized that foliar application of *β*-carotene in fenugreek may alleviate the harmful effect under drought stress conditions. The objective of this study was to observe the effects of *β*-carotene on morpho-physiological and biochemical parameters of fenugreek varieties under drought stress conditions.

## 2. Materials and methods

A pot experiment was conducted at the Old Botanical Garden of the University of Agriculture, Faisalabad, Pakistan, to study how a foliar spray of *β*-carotene affects the growth, biochemical makeup and physiological responses of Fenugreek under drought conditions. The experiment followed a completely randomized design (CRD) with three replications and involved 36 pots. Half of the pots were planted with Kasuri methi seeds and the other half were planted with a local variety. Both types of seeds were obtained from Faisalabad’s Ayub Agricultural Research Institute. Eight kilograms of soil were filled in each pot, and 18 seeds were sown per pot. One week after germination, the seedlings were thinned out and six uniform seedlings were left in each pot. Two drought stress levels were used: control and 50% field capacity. The drought stress treatment began 45 d after sowing, once the seedlings were well established. After two weeks of drought maintenance, three levels of *β*-carotene (0, 100, and 200 ppm) were applied as foliar sprays. To make the solution, 100 or 200 mg of *β*-carotene was added into the distilled water to make the volume of each up to 1 L, and 20% Tween-20 was added as a surfactant because of the lipophilic nature of *β*-carotene. The mixture was stirred constantly until the final volume reached 1 L. After four weeks of foliar application, the plants were harvested for morpho-physiological parameters.

### 2.1. Soil characteristics

The soil utilized in this experiment had an EC of 4 dS m^−1^ and a pH of 7.7, which indicates that it is somewhat alkaline. The soil texture comprised up to 42% sand, 17% silt, and 29–35% clay. We collected this soil from the research area and allowed it to air dry. Before we plant, we saturate the soil with water twice. These soil characteristics are crucial because high pH can impact the availability of micronutrients such as iron and zinc, and moderate EC can cause plants to experience osmotic stress in addition to dryness.

#### 2.1.1. Determination of field capacity

Pennypacker et al.^35^ provided a standard procedure for determining field capacity. The soil in each pot was first saturated with water and allowed to drain freely in order to calculate each soil's field capacity using the gravimetric method, resulting in 100% and 50% field capacity. The moisture content was then determined once a constant weight (100% FC) was attained. For treatments requiring 100% FC, the water lost through evapotranspiration had to be weighed and replaced in order to keep the pots' moisture content constant. By measuring the pots on a regular basis and adding only enough water to return them to 50% FC, 50% of the water holding capacity was maintained.

### 2.2 Harvesting

Three months after they were sown, the plants were harvested. For the purpose of recording morphological traits, two complete plants were carefully taken out of each pot, then their average was calculated. For physiological parameters, the fresh samples were collected, sealed in zipper bags, and kept at −20 °C in the medical freezer.

### 2.3 Morphological attributes

A digital weighing balance (Model; OHAUS, USA cooperation) was used to measure the fresh weight of the shoots and roots as soon as they were harvested. The shoot and root samples were first labeled and air-dried and then kept in an oven set between 65 and 70 °C for 14 d up to their constant weight. A digital balance was then used to determine their dry weight. These dried samples were later used for ion analysis. The length of each root and shoot was measured in centimeters using a measuring tape after harvesting.

### 2.4 Photosynthetic pigments

Chlorophyll and carotenoid contents were estimated using the protocol given by Arnon.^36^ For this purpose, 0.1 grams of fresh leaf was finely chopped and placed into small containers with 5 mL of 80% acetone. These samples were left in the room at 25 °C overnight to allow pigment extraction. A blank sample was prepared and run without plant sample known standard assay. After the incubation period, a spectrophotometer (Model: WE 721, WELab Instrument Limited) was used to measure the absorbance at 663, 645, and 480 nm. The pigment concentrations were calculated using the following formulas:

Chlorophyll a(mg/g FW)=[12.7×OD663−2.69×OD645]×W×V/1000

Chlorophyll b (mg/g FW)=[22.9×OD645−4.68×OD663] ×W×V/ 1000

Carotenoids (mg/g FW)=[OD480+0.114×OD663−0.638×OD645]/2500

### 2.5 Oxidative stress determinants

**Sample preparation**

To analyze oxidative stress determinants, 0.25 g of fresh plants were crushed in 3 mL of 0.5% trichloroacetic acid (TCA). This solution was transferred to an Eppendorf tube and at 12,000 rpm were centrifuged for 12 min. After being separated, the supernatant was kept in a different Eppendorf tube at −12 °C for further analysis.

#### 2.5.1. Malondialdehyde (MDA) content

The level of lipid peroxidation, indicated by the MDA content, was measured using the Cakmak and Horst’s^37^ method. One milliliter of the extract was mixed with 1 mL of 0.5% thiobarbituric acid (TBA) made in 20% TCA. The mixture was then intense in a water bath for 15 min at 95 °C, cooled on ice for another 15 min and readings were recorded at 532 and 600 nm using a spectrophotometer (Model: WE 721, WELab Instrument Limited).

#### 2.5.2. Hydrogen peroxide (H₂O₂) content

Hydrogen peroxide was calculated using the Velikova et al.^38^ technique. In a test tube, 0.5 mL of sample extract, K_3_PO_4_ buffer, and 1 mL of KI solution were added (prepared by dissolving 16.6 g of KI in 100 mL of distilled H_2_O). After vortexing, readings were recorded at 390 nm using the same spectrophotometer.

### 2.6 Organic osmolytes

#### 2.6.1. Total soluble proteins (TSP)

The approach was applied to measure the TSP. For each sample, 0.1 mL of extract was mixed with 5 mL of Bradford reagent in a test tube. After vortexing for 30 s at 595 nm, the absorbance was measured with a spectrophotometer (Mode: WE 721, WELab Instrument Limited). To prepare the Bradford reagent, 0.1 g of brilliant blue G-250 dye was dissolved in 50 mL of 95% ethanol, then mixed with 100 mL of 85% phosphoric acid. The solution was filtered by Whatman filter paper to reach the final volume of one liter.

#### 2.6.2. Total soluble sugars (TSS)

The Yoshida et al.^39^ protocol was used to estimate the TSS. Fresh leaf samples (0.1 g) were placed in test tubes with 10 mL of distilled water and heated in a water bath at 90 °C for 1 h. The solution was then diluted with 50 mL of distilled water. A 1.5 mL portion of this dilution was mixed with 5 mL of anthrone reagent (1 g of anthrone in 1 L of sulfuric acid) and heated again at 90 °C for 20 min. The readings were noted at 620 nm using the same spectrophotometer.

### 2.7 Enzymatic antioxidants

**Sample preparation**

A 0.25 g fresh leaf sample was crushed in 5 mL of cold phosphate buffer using an ice-cold mortar and pestle. The extract was then centrifuged for 15 min at 12,000 rpm, and the supernatant was obtained for enzyme activity measurements. A blank sample was prepared without plant sample extract; however, all the other solutions were mixed and run at the same absorbance known for standard calibration curves (R^2^ ≥ 0.99).

#### 2.7.1. Superoxide dismutase (SOD)

SOD activity was estimated using the method of Spitz and Oberley.^40^ A cuvette was filled with the following: 400 µL distilled water, 250 µL phosphate buffer, 100 µL L-methionine, as well as Triton-X, 50 µL NBT, plant extract and riboflavin. The cuvette was kept under light for 15 min, and the absorbance was measured at 560 nm.

#### 2.7.2. Peroxidase (POD)

The peroxidase activity was measured using Chance and Maehly's approach (1955). In a cuvette, 750  µL of K_3_PO_4_, 100  µL of guaiacol and H₂O₂, and 50  µL of plant extract were filled. The absorbance was recorded at 470 nm at 0, 30, 60, and 90 s.

#### 2.7.3. Catalase (CAT)

The CAT activity was also examined by the Chance and Maehly^41^ method. A cuvette containing 1.9  mL of K_3_PO_4_ buffer, 1  mL of H₂O₂ and 0.1  mL of plant extract was used. The absorbance was recorded at 240 nm at 30-second intervals up to 90 s.

### 2.8 Non-enzymatic antioxidants

#### 2.8.1. Ascorbic acid content

The approach of Mukherjee and Choudhuri^42^ was applied. After grinding a 0.25 g fresh leaf, in 1 mL of 6% TCA, the material was centrifuged for 12 min at 12,000 rpm. Two milliliters of the extract, 1 mL of 2% dinitrophenylhydrazine and a drop of 10% thiourea in 70% ethanol, were combined. In a water bath, the test tubes were heated to 90 °C for 15 min. Five milliliters of 80% sulfuric acid were added after cooling, and the absorbance at 530 nm was measured.

#### 2.8.2. Anthocyanin content

Using the Stark and Wray^43^ method, 0.1 g of fenugreek leaf was chopped and soaked in 5 mL of acidified methanol (prepared by mixing 160 mL of methanol with 1 mL of HCl). The mixture was heated at 50 °C for 1 h and the absorbance was recorded at 535 nm.

#### 2.8.3. Flavonoid content

Flavonoids were measured using Kim et al.^44^ method. A 0.1 g fenugreek leaf sample was soaked in 80% acetone overnight in the dark. The next day, 1 mL of this extract was added to 4 mL of distilled H_2_O, 0.6 mL of 5% NaNO₂, and after 5 min, 0.5 mL of 10% AlCl₃. After 1 min, 2 mL of 1 M NaOH and 2.4 mL of distilled water were added. Reading was noted at 510 nm.

### 2.9 Statistical analysis

A completely randomized design was used to design the experiment with three replications. Statistical significance was determined using CoStat software. Using Statistix 8.1 software, three-factorial analysis of variance was carried out, and Tukey's test was used to compare the means. R-studio (v4.3.3), a statistical tool, was utilized for creating the heatmap with a dendrogram and the Pearson correlation coefficient, Originpro (V2024) was used to make PCA analysis, Microsoft Excel (Version 2016) was used to generate graphs.

## 3. Results

### 3.1. Morphological parameters

The morphological parameters of the fenugreek varieties were significantly (*p* ≤ 0.05) affected by drought and *β*-carotene treatment. Drought stress decreases the shoot fresh weight by 35.02% and 58.04%, shoot dry weight 42.5% and 50.5%, and shoot length by 17.12% and 17.14% in Kasuri and Local as compared to control. By the application of 100 ppm *β*-carotene, the shoot fresh weight increased by 71.8% and 37.6%, the shoot dry weight increased by 83.8% and 48.5%, and the shoot length increased by 20.7% and 21.5% in Kasuri and Local under drought stress conditions. Under 200  ppm *β*-carotene application, shoot fresh weight increased by 95.06% and 66.7%, shoot dry weight increased by 182.8% and 93.1%, and shoot length increased by 49.6% and 44.5% in Kasuri and local plants under drought stress conditions. Compared with the control, drought stress increased the fresh weight of the roots by 133% and 26.2%, the dry weight of the roots by 64.8% and 23.3%, and the root length by 109.1% and 13.4% in Kasuri and Local as compared to control. By the application of 100 ppm *β*-carotene, the root fresh weight further increased by 47.4% and 14.7%, root dry weight 53.1% and 38.2%, and root length 48.0% and 23.8% in Kasuri and Local under drought stress conditions. Under 200 ppm *β*-carotene application root fresh weight increased by 79.1% and 37.0%, root dry weight 118.0% and 102.2%, the root length increased by 89.5% and 46.2% in Kasuri methi and Local under drought stress conditions, respectively (Table 1).

**Table 1.** Mean square value of *β*-carotene and drought stress on morphological parameters.

Varieties	Stress	Treatment	RFW	RDW	RL	SDW	SFW	SL
**Kasuri methi**	**Control**	**BC 0 ppm**	0.14 ± 0.015ef	0.03 ± 0.005cd	3.556 ± 0.568d	0.596 ± 0.021ac	3.719 ± 0.27bd	24.43 ± 2.602bc
		**BC 100 ppm**	0.184 ± 0.010df	0.04 ± 0.005bd	6.183 ± 0.348bd	0.674 ± 0.019ab	4.628 ± 0.378ab	29.15 ± 0.433ab
		**BC 200 ppm**	0.22 ± 0.013ce	0.054 ± 0.003b	8.48 ± 0.35ab	0.789 ± 0.040a	5.313 ± 0.491a	32.5 ± 0.894a
	**Drought** **50% FC**	**BC 0 ppm**	0.176 ± 0.005df	0.037 ± 0.004bd	7.44 ± 0.663ac	0.343 ± 0.029df	2.417 ± 0.124de	20.25 ± 1.01ce
		**BC 100 ppm**	0.260 ± 0.022ad	0.056 ± 0.006b	9.216 ± 0.88ab	0.509 ± 0.0403bd	3.326 ± 0.1908bd	24.45 ± 1.856bc
		**BC 200 ppm**	0.316 ± 0.009ab	0.080 ± 0.005a	10.88 ± 0.728a	0.66 ± 0.026ac	4.03 ± 0.202ac	29.266 ± 1.112ab
**Local**	**Control**	**BC 0 ppm**	0.106 ± 0.012f	0.018 ± 0.004d	4.053 ± 0.360cd	0.333 ± 0.0318df	3.210 ± 0.265bd	17.133 ± 0.169de
		**BC 100 ppm**	0.208 ± 0.004ce	0.037 ± 0.002bd	6.256 ± 0.538bd	0.494 ± 0.038be	3.8633 ± 0.370ad	22.606 ± 1.224cd
		**BC 200 ppm**	0.237 ± 0.0173bd	0.043 ± 0.003bc	6.9 ± 0.987bd	0.598 ± 0.037ac	4.55 ± 0.326ab	25.136 ± 0.586bc
	**Drought** **50% FC**	**BC 0 ppm**	0.247 ± 0.015bd	0.029 ± 0.0014cd	4.6 ± 0.598cd	0.165 ± 0.0617f	1.35 ± 0.312e	14.196 ± 0.736e
		**BC 100 ppm**	0.283 ± 0.034ac	0.041 ± 0.004bd	6.81 ± 0.716bd	0.303 ± 0.016ef	2.32 ± 0.2015de	17.25 ± 0.433de
		**BC 200 ppm**	0.338 ± 0.019a	0.06 ± 0.005ab	8.72 ± 0.601ab	0.466 ± 0.0612ce	2.633 ± 0.280ce	21.24 ± 0.578cd

Data are presented as means ± standard error based on three replicates. Fenugreek varieties assigned the same letter for a given parameter did not differ significantly at the *p* ≤ 0.05 level. Fenugreek has the varieties V1 Kasuri methi and V2 Local, which have different drought stress levels (control and 50% FC) and β-carotene (BC) concentrations (control, 100 and 200 ppm). Letters indicate significant differences at the *p* ≤ 0.05 level among all treatment combinations (three-way ANOVA, Tukey’s HSD), and the same letter for a given parameter indicates non-significant differences.

### 3.2. Photosynthetic pigments

**Figure 1.**
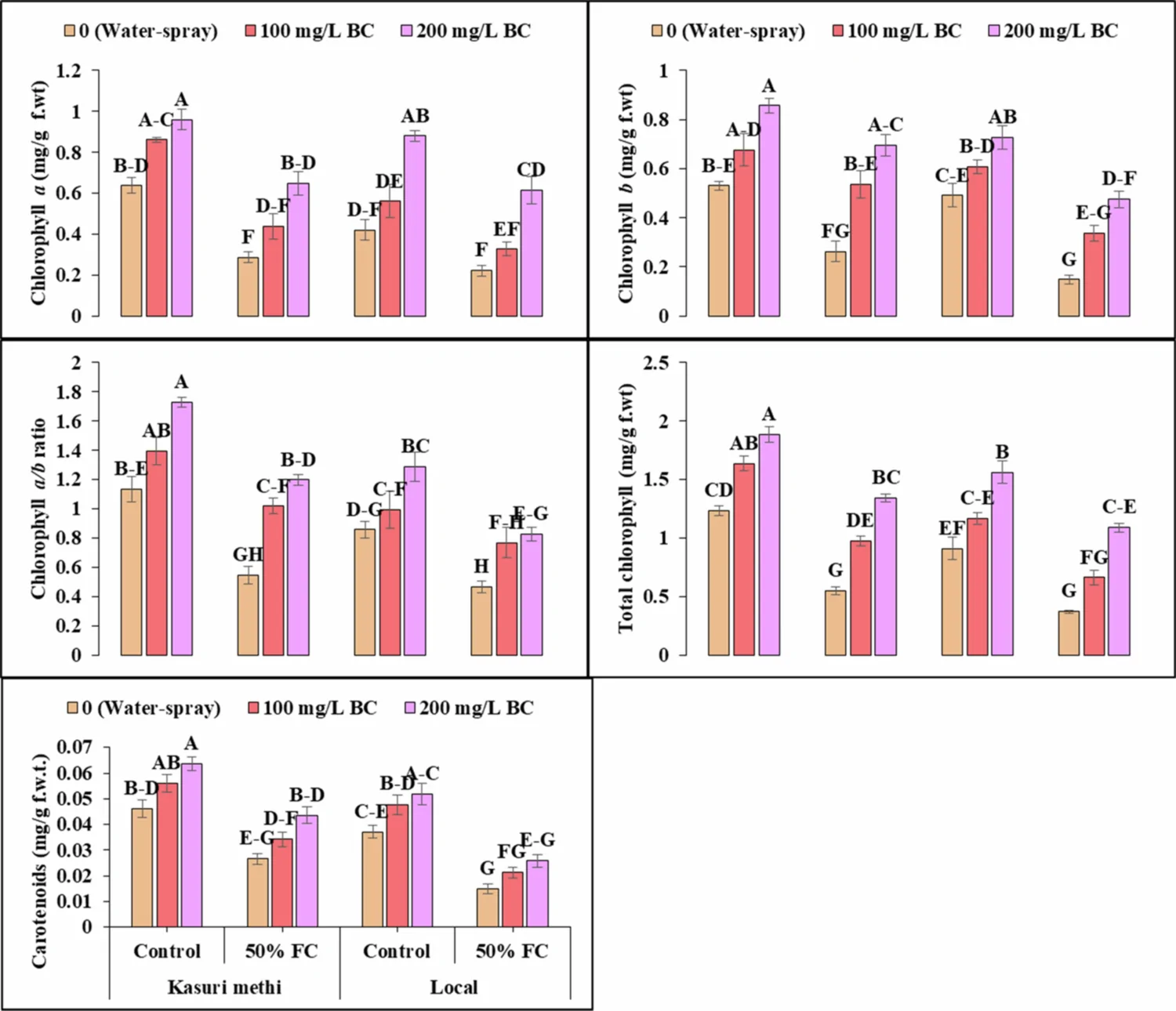
Effect of *β*-carotene on chlorophyll *a*, chlorophyll *b*, chlorophyll *a/b* ratio total chlorophyll and carotenoids under drought stress. Fenugreek has varieties V1 Kasuri methi and V2 Local, drought stress level (control and 50% FC), and *β*-carotene (BC) (control, 100, and 200 ppm). Letters above bars indicate significant differences at the *p* ≤ 0.05 level among all treatment combinations (three-way ANOVA, Tukey’s HSD) and the same letter for a given parameter show non-significant differences.

Photosynthetic pigments of fenugreek varieties were significantly (*p* ≤ 0.05) affected by drought as well as *β*-carotene. Drought stress decreases the Chl. *a* by 54.9% and 47.1%, Chl. *b* 50.4% and 69.8% and Chl. *a/b* 51.7% and 45.7% in Kasuri and Local as compared to control. By the application of 100  ppm *β*-carotene, the Chl. *a* increased by 52.3% and 46.6%, Chl. *b* 104.4% and 127.2% and Chl. *a/b* 85.9% and 65.07% in Kasuri and Local. Under 200 ppm *β*-carotene application, Chl. *a* increased by 125.5% and 176.2%, Chl. *b* 164.9% and 221.6%, Chl. *a/b* 118.9% and 77.4% in the Kasuri methi and Local under drought stress conditions. Drought stress decreases the total Chl. by 55.4% and 59.3%, and carotenoids 42.1% and 59.3% in Kasuri and Local as compared to control. By the application of 100  ppm *β*-carotene, the total Chl. increased by 77.2% and 78.8%, and carotenoids 28.08% and 41.8% in Kasuri and Local under drought stress conditions. Under 200 ppm *β*-carotene application total Chl. increased by 194.3% and 144.3%, respectively, and those of carotenoids increased by 71.6% and 63%, in Kasuri methi and Local under drought stress conditions (Figure 1).

### 3.3. Reactive oxygen species, stress indicator, and leaf ascorbic acid

**Figure 2.**
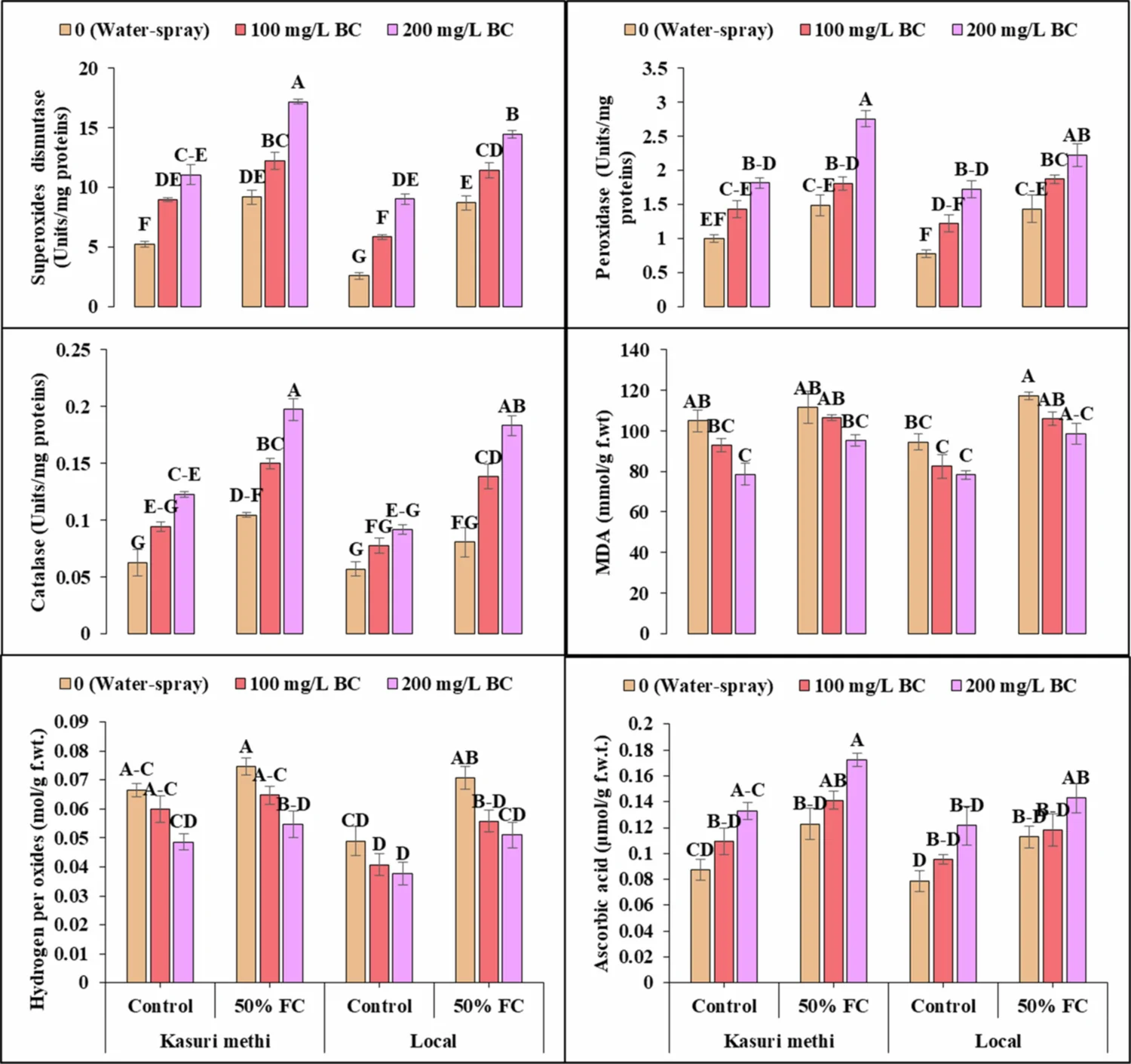
Effect of *β*-carotene on superoxide dismutase, peroxidase, catalase, hydrogen peroxides, MDA, and ascorbic acid under drought stress. Fenugreek has varieties V1 Kasuri methi and V2 Local, drought stress level (control and 50% FC), and *β*-carotene (BC) (control, 100 and 200  ppm). The letters above the bars indicate significant differences at the *p* ≤ 0.05 level among all the treatment combinations (three-way ANOVA, Tukey’s HSD), and the same letter for a given parameter show non-significant differences.

Statistical analysis showed that the reactive oxygen species and stress indicators of fenugreek were significantly impacted by both drought as well as *β*-carotene treatment. Drought stress increased the MDA content by 6.35% and 24.2%, the hydrogen peroxides 12.05% and 44.2%, and the leaf ascorbic acid content by 41.04% and 43.0% in Kasuri and Local as compared to control. By the application of 100 ppm *β*-carotene, the MDA decreased by 4.6% and 9.5%, the hydrogen peroxide 13.2% and 21.0%, while leaf ascorbic acid content increased by 14.7% and 4.8% in Kasuri and Local under drought stress conditions. Under 200 ppm *β*-carotene application, the MDA content decreased by 14.7% and 15.8%, the hydrogen peroxides 26.6% and 27.8%, while leaf ascorbic acid content increased by 40.01% and 26.7% in Kasuri methi and Local under drought stress condition (Figure 2).

### 3.4. Enzymatic antioxidants

Statistical analysis showed that the enzymatic antioxidants activities of fenugreek were significantly impacted by drought and *β*-carotene treatment. Drought stress increased the superoxide dismutase activity by 75.3% and 240.5%, peroxidase 49.7% and 86.2%, and catalase 66.8% and 41.5% in Kasuri and Local as compared to control. By the application of 100 ppm *β*-carotene, the superoxide dismutase activity increased by 32.7% and 31.1%, peroxidase 21.5% and 30.3%, and catalase 43.4% and 72.19% in Kasuri and Local under drought stress conditions. Under 200 ppm *β*-carotene application, superoxide dismutase increased by 86.7% and 65.7%, peroxidase 85.4% and 54.8%, and catalase 89.0% and 128.3% in Kasuri methi and Local under drought stress conditions (Figure 2).

### 3.5. Non-enzymatic antioxidants

**Figure 3.**
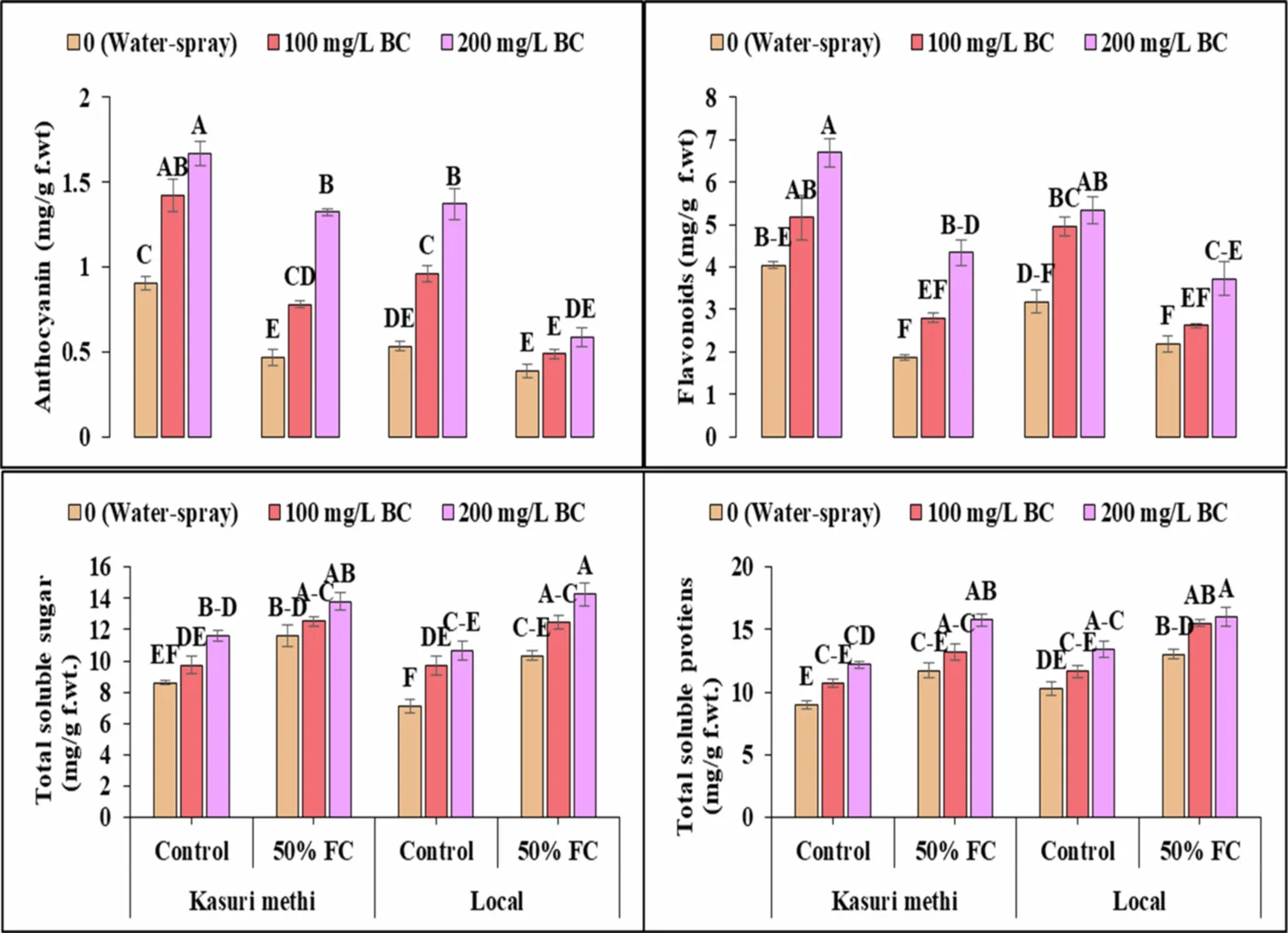
Effect of *β*-carotene on anthocyanin, flavonoids, total soluble sugar and total soluble proteins under drought stress. Fenugreek has varieties V1 Kasuri methi and V2 Local, drought stress level (control and 50% FC), and *β*-carotene (BC) (control, 100 and 200 ppm). The letters above the bars indicate significant differences at the *p* ≤ 0.05 level among all the treatment combinations (three-way ANOVA, Tukey’s HSD), and the same letter for a given parameter show non-significant differences.

Statistical data revealed that the non-enzymatic antioxidants activities and osmolytes of fenugreek were significantly affected by drought stress and *β*-carotene treatment. Drought stress decreases the anthocyanin by 48.3% and 27.5%, flavonoids 53.8% and 31.2%, increased total soluble sugar by 34.6% and 45.2%, and the total soluble protein content by 29.8% and 26.3% in the Kasuri and Local as compared to control. By the application of 100 ppm *β*-carotene, the anthocyanin increased by 67.3% and 26.2%, flavonoids 50.3% and 19.6% and total soluble sugar 8.03% and 20.6%, and in total soluble protein 12.6% and 19.04% in Kasuri and Local under drought stress conditions. Under 200 ppm *β*-carotene application, anthocyanin increased by 183.9% and 51.8%, flavonoids 132.7% and 70.4%, total soluble sugar by 19.01% and 37.8% and total soluble protein 34.2% and 22.9% in Kasuri methi and Local under drought stress condition (Figure 3).

### 3.6. Inorganic ions

Statistical data showed that the inorganic ions of fenugreek were significantly impacted by drought stress and *β*-carotene treatment. Drought stress decreases the shoot K^+^ by 21.4% and 31.4%, shoot Na^+^ 33.3% and 11.1%, and the shoot Ca^2+^ 35.3% and 44.5% in Kasuri and Local as compared to control. By the application of 100 ppm *β*-carotene, the shoot K^+^ increased by 40.9% and 16%, the shoot Na^+^ 22.5% and 13.3%, and the shoot Ca^2+^ 78.5% and 43.4% in Kasuri and Local under drought stress conditions, respectively. Under 200  ppm *β*-carotene application, shoot K^+^ increased by 54.5% and 48.1%, shoot Na^+^ 40% and 43.3%, and shoot Ca^2+^ 102.38% and 73.91% in Kasuri methi and Local under drought stress condition. Drought stress increased the root K^+^ by 4.5% and 20.7%, root Na^+^ by 25.8% and 42.7%, and root Ca^2+^ by 20.2% and 20.9% in Kasuri and Local as compared to control. By the application of 100  ppm *β*-carotene, the root K^+^ increased by 19% and 14.2%, root Na^+^ 6.3% and 10.2% and root Ca^2+^ 29% and 20.5% in Kasuri and local plants under drought stress conditions. Under 200  ppm *β*-carotene application, root K^+^ increased by 47.6% and 45.2%, root Na^+^ increased by 21.9% and 25.5%, and root Ca^2+^ increased by 41.8% and 30.8% in Kasuri methi and Local under drought stress conditions (Table 2).

**Table 2.** Mean Square Value of *β*-carotene and drought stress on ionic parameters.

Varieties	Stress	Treatment	Shoot K^+^	Root K^+^	Shoot Na^+^	Root Na^+^	Shoot Ca^2+^	Root Ca^2+^
**Kasuri methi**	**Control**	**BC 0 ppm**	9.33 ± 1.203cd	14.666 ± 1.454be	10 ± 1.156ef	18.667 ± 0.337bd	10.833 ± 0.834cd	11.5 ± 1.042ef
		**BC 100 ppm**	12 ± 1.156bd	23.666 ± 0.882ab	8.666 ± 1.203df	15.833 ± 0.441ab	12.667 ± 0.667bd	14.833 ± 0.441de
		**BC 200 ppm**	15 ± 0.578ac	27.666 ± 0.882a	5.667 ± 0.882ce	12.8233 ± 0.441a	15 ± 0.578b	17.667 ± 0.337bd
	**Drought** **50% FC**	**BC 0 ppm**	7.333 ± 1.203bd	14 ± 1.156f	13.333 ± 0.882df	23.5 ± 1.758df	7 ± 0.578bd	9.166 ± 0.1668bd
		**BC 100 ppm**	10.333 ± 0.882ab	16.666 ± 2.406ef	10.333 ± 0.882ad	22 ± 0.578bd	12.5 ± 1.758b	11.833 ± 0.441ac
		**BC 200 ppm**	11.333 ± 0.882a	20.667 ± 0.882bd	8 ± 1.256ab	18.333 ± 0.337ac	14.166 ± 0.441a	13 ± 0.578ab
**Local**	**Control**	**BC 0 ppm**	12.333 ± 1.203d	17.666 ± 1.203df	9 ± 0.5780f	16 ± 1.529bd	13.833 ± 0.929d	14.333 ± 0.727f
		**BC 100 ppm**	15 ± 1.734bd	24 ± 1.529bc	7.333 ± 0.667ce	13 ± 1.5293ad	16 ± 0.578bd	18 ± 0.289de
		**BC 200 ppm**	18 ± 1.529bd	26.333 ± 2.336ab	5.667 ± 0.883bd	10.333 ± 1.454ab	18.5 ± 1.258bc	19.167 ± 1.643ce
	**Drought** **50% FC**	**BC 0 ppm**	8.333 ± 0.882bd	14 ± 1.1561f	10 ± 1.156bd	22.8333 ± 0.4415e	7.667 ± 1.454cd	11.33 ± 0.667ce
		**BC 100 ppm**	9.667 ± 0.882bd	16 ± 2.084ef	8.667 ± 1.203ac	20.5 ± 0.289de	11 ± 1.156bd	13.667 ± 0.883ac
		**BC 200 ppm**	12.333 ± 0.862ab	20.33 ± 0.883ce	5.667 ± 0.883a	17 ± 0.867ce	13.33 ± 0.883ab	14.833 ± 0.8343a

Data are presented as means ± standard error based on three replicates. Fenugreek varieties assigned the same letter for a given parameter did not differ significantly at the *p* ≤ 0.05 level. Fenugreek have varieties V1 Kasuri methi and V2 Local, drought stress levels (control and 50% FC), and β-carotene (BC) (control, 100 and 200 ppm). Letters indicate significant differences at the *p* ≤ 0.05 level among all treatment combinations (three-way ANOVA, Tukey’s HSD), and the same letter for a given parameter show non-significant differences.

### 3.7. Heatmap with dendrogram

**Figure 4.**
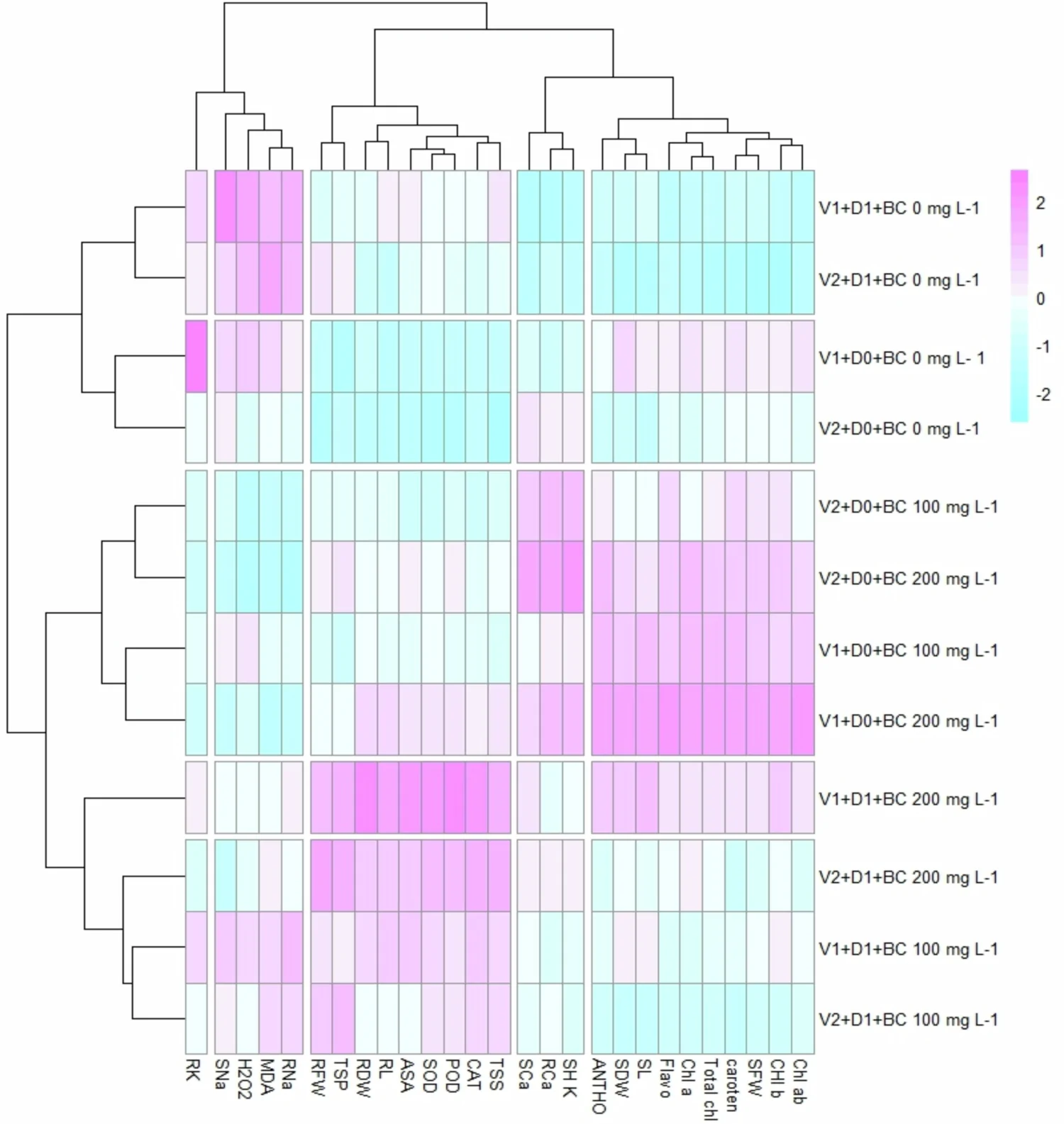
Heatmap analysis with dendrogram between various morpho-biochemical, physiological and ionic attributes of fenugreek exposed to drought stress and *β*-carotene foliar application. V1 = Kasuri methi, V2 = Local; D0 = control, and D1 = 50% FC and *β*-carotene (BC) (control, 100  and 200 ppm).

A dendrogram was included in a two-way heatmap to show the role of *β*-carotene in fenugreek under drought stress (Figure 4). The standardized results for every plant attributed under all the stress conditions and treatments were illustrated in the cluster heat map as colored squares to allow comparison of the treatments and stress on the specific features of the plants. For several observations, the lavender color showed positive correlation while lite blue color showed a highly negative correlation. Five categories have been identified in the heatmap. In the first category, chlorophyll *a/b*, Chl. *a*, *b*, shoot fresh and dry weight, shoot length, carotenoids, total chlorophyll, anthocyanin, and flavonoids. Strong positive correlations exist between these metrics at D0 (no stress) and 200 mg L^−1^ *β*-carotene and strong negatively correlated under D1 (50% FC) and 0 mg L^−1^ *β*-carotene treatment application. This group demonstrated that the *β*-carotene improved the levels of total chlorophyll, carotenoids, shoot fresh and dry weight, anthocyanins, and flavonoids, which minimized the negative impacts of oxidation caused by drought stress. The second category comprised root and shoot Ca^2+^ and shoot K^+^, which showed a strong positive effect under D0 (not stress) and 200 mg L^−1^ *β*-carotene while exhibited a negative effect under D1 (50% FC) and 0 mg L^−1^ *β*-carotene treatment application among both varieties. The third group contained root dry and fresh weight, peroxidase (POD), root length, total soluble solids (TSS), superoxide dismutase (SOD), catalase (CAT), leaf AsA, and total total soluble protein (TSP). These indices revealed a strong positive correlation at D1 (50% FC) and 200 mg L^−1^ *β*-carotene application, while strong negatively correlated under control conditions under D0 (no stress) and 0 mg L^−1^ *β*-carotene application. The fourth group contained root and shoot Na^+^, H_2_O_2_, and MDA. The activities of reactive oxygen species and stress indicator showed a substantial positive correlation at D1 (50% FC) and 0 mg L^−1^ *β*-carotene application, and a negative association at D0 (no stress) and 200 mg L^−1^ *β*-carotene treatment application. These findings showed that Na^+^ accumulation and reactive oxygen species and stress indicators of fenugreek varieties were alleviated in plants that were treated with *β*-carotene. The fifth group contained shoot K^+^, that is positively correlated at D0 (no stress) and 0 mg L^−1^ *β*-carotene treatment, and negative correlation at D0 (no stress) and 200 mg L^−1^ *β*-carotene treatment. This illustration showed that by regulating osmotic adjustment, membrane integrity, and enzyme activation, ionic attributes regulate physiological activities. In particular, shoot and root K^+^ and Ca^2+^ improve water retention and photosynthetic activity, while Na⁺ accumulation during drought inhibits both features. For morpho-physiological and biochemical parameters more effective results were observed in those plants that were treated with *β*-carotene foliar application (Figure 4).

### 3.8. Correlation analysis

**Figure 5.**
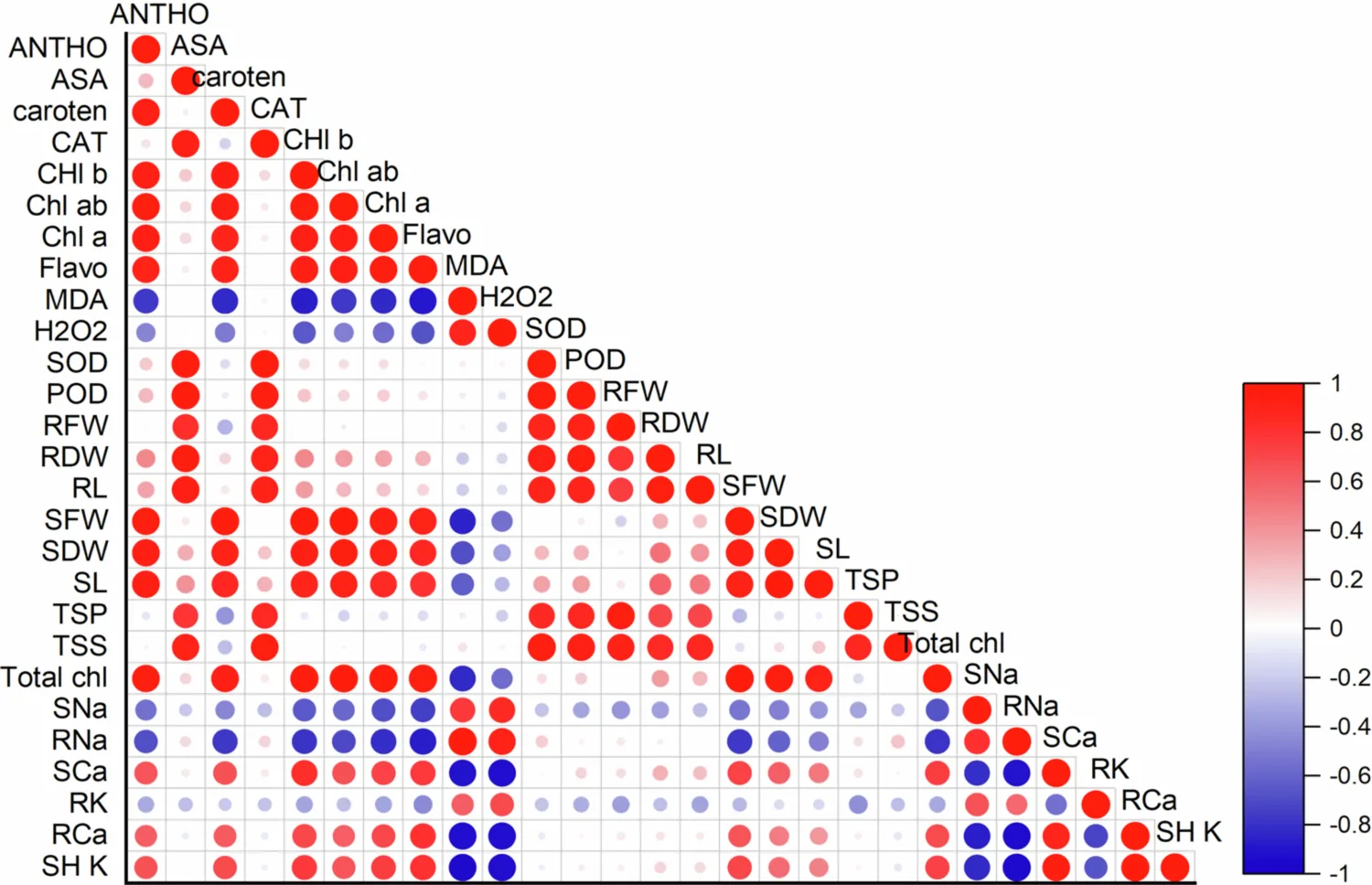
Pearson correlation analysis between different morphological parameters, photosynthetic pigments, biochemical and ionic attributes of fenugreek varieties by foliar application of *β*-carotene under drought stress.

According to the results of the Pearson correlation analysis, the biochemical and morpho-physiological parameters of both fenugreek varieties showed a substantial association (Figure 5). A significant positive correlation was observed between most indicators, including morphological attributes and photosynthetic pigments such as dry and fresh biomass of the shoot, shoot length, carotenoids, and total chlorophyll. Similarly, there is a positive association found between the dry and fresh weight of root, root length, CAT, POD, SOD, TSP, leaf AsA and TSS. However, stress indicators and oxidants such as MDA and H_2_O_2_, and root and shoot Na^+^ exhibited inverse relationships with flavonoids, anthocyanins, root and shoot K^+^, and Ca^2+^ (Figure 5).

### 3.9. PCA analysis

**Figure 6.**
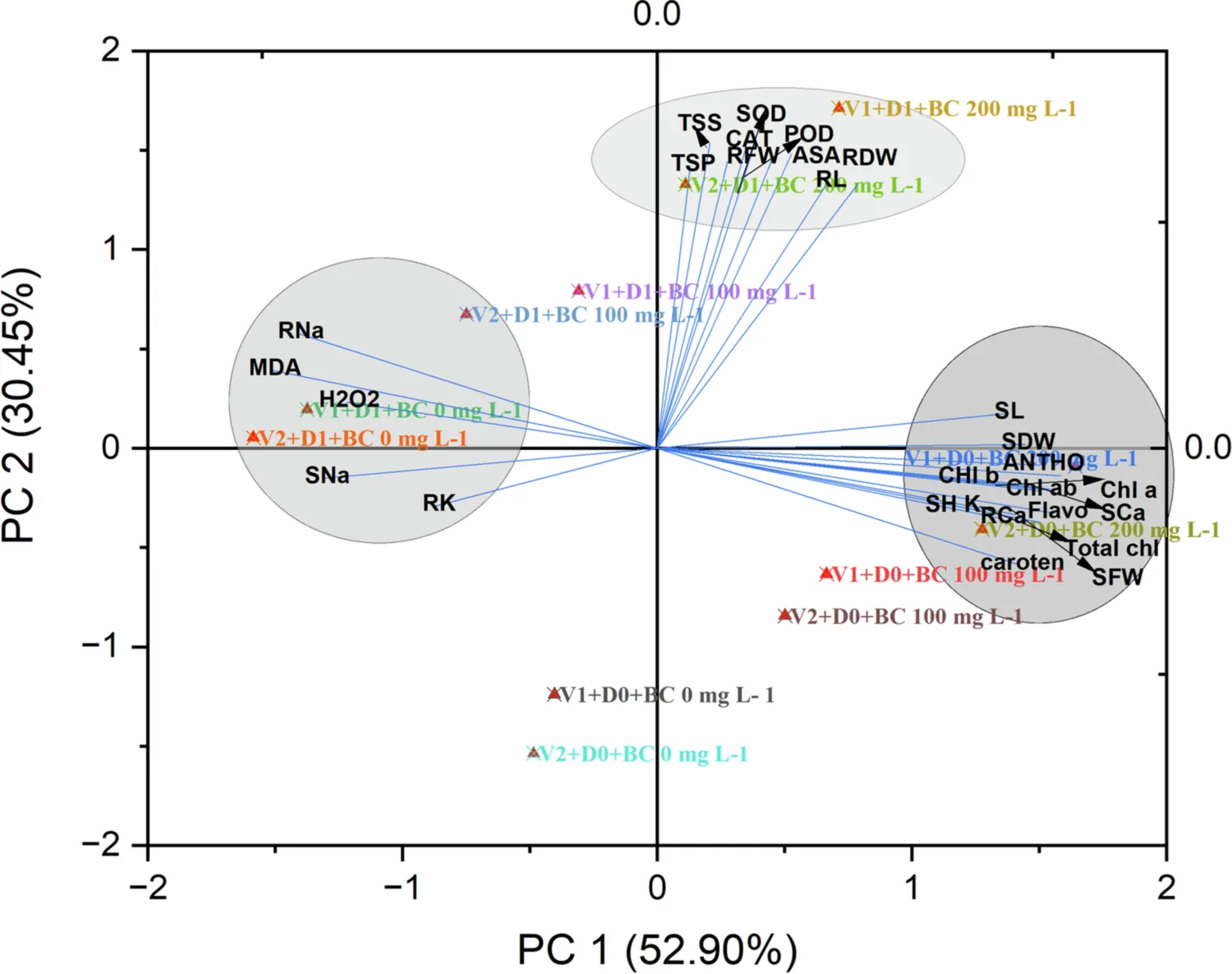
Principal component analysis between different morphological parameters, photosynthetic pigments, and biochemical and ionic attributes of fenugreek varieties by foliar application of *β*-carotene under drought stress. V1 = Kasuri methi, V2 = Local; D0 = control, and D1 = 50% FC and *β*-carotene (BC) (control, 100  and 200 ppm).

Principal component analysis (PCA) demonstrated that PCA1 (52.90%) and PCA2 (30.45%) exhibited the morphological, photosynthetic, antioxidants, and organic osmolytes of both fenugreeks. There was a strong correlation found between different photosynthetic and morphological attributes with non-enzymatic antioxidants (anthocyanins and flavonoids), and shoot and root fresh K^+^ and Ca^2+^. However, there is a close relationship among root fresh and dry weight, root length, enzymatic antioxidants, organic osmolytes as well as leaf ascorbic acid. At the same time, H_2_O_2_ and MDA as well as shoot and root fresh Na^+^, showed a close relationship among themselves. In our study, *β*-carotene treatment exhibited greater efficacy in reducing drought stress by enhancing morphological characteristics such as shoot length, shoot fresh and dry weight, and photosynthetic pigments, enzymatic and non-enzymatic antioxidants, as well as organic osmolytes, while lessening the adverse effects of reactive oxygen species among both fenugreek varieties (Figure 6).

## 4. Discussion

Drought stress substantially impairs the growth and productivity of fenugreek, as reported in recent studies.^45^ At the biochemical level, water deficit disrupts cellular redox homeostasis and promotes the excessive generation of reactive oxygen species (ROS), resulting in increased accumulation of oxidative stress markers such as MDA and H₂O₂.^16^ ^,^ ^46^ The elevation of MDA reflects enhanced membrane lipid peroxidation, whereas increased H₂O₂ indicates severe oxidative pressure within plant tissues. In response, fenugreek activates its antioxidant defense system, as demonstrated by the enhanced activities of key antioxidant enzymes that detoxify ROS and limit cellular injury.^47^ These interconnected physiological and biochemical responses represent an important adaptive mechanism through which fenugreek attempts to maintain cellular integrity and metabolic function under limited water availability. Recent evidence further indicates that *β*-carotene can contribute to improved drought tolerance in fenugreek. *β*-Carotene is an essential non-enzymatic antioxidant that scavenge ROS generated under drought conditions. Through the restriction of oxidative damage, *β*-carotene may reduce membrane lipid peroxidation, and preserve cellular structures. Therefore, the *β*-carotene-mediated enhancement of antioxidant protection may partly explain the improved physiological performance and greater tolerance of fenugreek plants exposed to drought stress. The better water relations in *β*-carotene treated plants might also be linked to better protection of cells and signaling that is responsive to stress. Stabilization of cellular membranes increases the water in the cells and lessens injury caused by dehydration. Moreover, carotenoid-signaling molecules are associated with other hormonal pathways involved in stomatal regulation and water-use efficiency. These responses help cells to survive under water shortage and maintain cellular activity, which contributes to enhanced drought tolerance ().

The present study demonstrated that drought stress adversely affects the morphological performance of fenugreek, as evidenced by pronounced reductions in shoot growth and related morphological traits. These findings are consistent with previous reports on wheat,^48^ maize,^49^ and fenugreek,^50^ in which drought-induced growth inhibition was primarily attributed to reduced cellular water content and loss of turgor pressure. Such changes restrict cell expansion and division, ultimately suppressing overall plant development.^51^ In contrast to shoot growth, the enhancement of root length under water-deficit conditions may represent an important drought-avoidance strategy. Greater root elongation enables plants to explore deeper soil layers and access otherwise unavailable water reserves, thereby improving water acquisition and sustaining plant survival under drought stress.^52^ The application of *β*-carotene considerably alleviated drought-induced morphological damage and improved the growth performance of fenugreek. Similar beneficial effects of *β*-carotene on plant morphology have been reported in tomato,^53^ olive,^54^ and spinach.^55^ The improvement in growth traits may be associated with the strong antioxidant properties of *β*-carotene, which enable it to quench reactive oxygen species and limit oxidative injury under water-deficit conditions. Moreover, *β*-carotene contributes to the stabilization of chloroplast and cellular membranes, thereby maintaining essential physiological and metabolic processes. Consequently, reduced oxidative damage and improved membrane stability may support continued cell division, elongation, and biomass accumulation under drought stress.^56^ These findings indicate that exogenous *β*-carotene application can enhance the morphological resilience of fenugreek by strengthening antioxidant protection and preserving cellular functionality during water limitation.

This decrease in membrane damage may be explained by the protection role of *β*-carotene on the lipid peroxidation. The cell membrane is very sensitive to oxidative attack because it contains high concentration of unsaturated fatty acids. Too much ROS causes excessive membrane lipid oxidation, which leads to increased membrane permeability and disruption of the compartmentalization of cells. By scavenging ROS and boosting antioxidant defenses, *β*-carotene helps maintain membrane stability and is responsible for the transport of water, nutrients, and metabolites in a proper manner within plant tissues. Under drought conditions, maintaining the integrity of the membranes is crucial for maintaining physiological processes.^57^

The findings of the present study confirm that drought stress significantly reduces the photosynthetic pigments of fenugreek by decreasing the chlorophyll and carotenoid contents. This observation is consistent with previous studies on olive,^54^ tomato,^53^ and wheat,^48^ which also reported substantial losses of photosynthetic pigments under drought conditions. This decline may be attributed to impaired chloroplast function and accelerated chlorophyll degradation, as drought-induced water deficits promote stomatal closure, restrict CO₂ availability, and ultimately disrupt photosynthetic pigments.^55^ Conversely, the foliar application of *β*-carotene effectively increased chlorophyll and carotenoid contents under drought stress. Similar responses have been reported in tricolor amaranth,^58^ maize,^59^ and fenugreek.^60^ As a potent antioxidant, *β*-carotene protects the chlorophyll pigments by stabilizing chloroplast membranes and safeguarding pigments against oxidative damage caused by drought-induced reactive oxygen species. This protective action limits pigment degradation and supports pigment biosynthesis under water-deficit conditions.^54^

The maintenance of the chlorophyll content after the application of *β*-carotene is probably linked with decreased oxidation of photosynthetic pigments. Under drought conditions, an excess ROS causes the rapid degradation of chlorophylls and disrupt electron transport in chloroplasts. The *β*-carotene neutralizes ROS and protects the pigment–protein complexes and chloroplast membranes, thus ensuring efficient light harvesting and electron transport. As a result, drought stress does not significantly affect the photosynthetic pigment biosynthesis, which helps preserve carbohydrates' synthesis and biomass production.^61^

The present study showed that drought stress reduced the accumulation of secondary metabolites, including anthocyanins and flavonoids. These findings are consistent with previous studies on spinach^55^ and fenugreek,^62^ which reported the disruption of secondary metabolism under water-deficit conditions. This decline may result from reduced carbon assimilation, limited availability of metabolic precursors, and enhanced oxidative stress, which collectively restrict the biosynthesis and accumulation of protective compounds such as anthocyanins and flavonoids.^54^ In contrast, foliar application of *β*-carotene enhanced the levels of these metabolites, which is in agreement with findings in olive,^54^ tomato,^53^ and amaranth.^58^ By scavenging drought-induced ROS, *β*-carotene limits oxidative damage and creates a favorable cellular environment for the biosynthesis of flavonoids and anthocyanins, which further strengthen plant defense under drought stress.^58^ The present study also demonstrated that drought stress increased the ASA, TSP, and TSS contents. Similar responses have been reported in wheat,^48^ maize,^63^ and fenugreek.^64^ These compounds function as important antioxidants and osmoprotectants by maintaining cellular osmotic balance, protecting membranes, and scavenging ROS, thereby improving tolerance to oxidative and osmotic stress under water-deficit conditions.^65^ Consistent with the present findings, *β*-carotene application further increased the ASA, TSP, and TSS contents in tomato,^53^ fenugreek,^66^ and other higher plants.^67^ This response may be attributed to *β*-carotene-mediated regulation of stress metabolism, improved oxidative protection, and enhanced osmolyte accumulation during drought stress.^67^

The present study confirmed that drought stress significantly increased the activities of enzymatic antioxidants, including POD, CAT, and SOD. Similar increases in POD, SOD, and CAT activities under water-deficit conditions have been reported in fenugreek,^68^ pea,^69^ and spinach.^70^ The enhancement of these antioxidant enzymes represents an important defensive response against drought-induced oxidative stress. SOD converts superoxide radicals into H₂O₂, whereas CAT and POD subsequently detoxify H₂O₂, thereby limiting oxidative damage to cellular components.^71^ ^,^ ^72^ Consistent with the present findings, *β*-carotene application further enhanced the activities of POD, CAT, and SOD in spring wheat,^73^ and olive.^54^ As an important non-enzymatic antioxidant, *β*-carotene directly quenches reactive oxygen species and protects cellular membranes from oxidative injury. Its application may also strengthen the enzymatic antioxidant system, thereby improving ROS detoxification and enhancing plant tolerance to drought stress.^55^

The improved antioxidant enzyme activity in *β*-carotene treated plants may be attributed to the activation of defense mechanisms that detoxify ROS. Superoxide dismutase, catalase, peroxidase and ascorbate peroxidase are the main antioxidant enzymes that transform harmful ROS molecules to less harmful molecules. These enzymes are more active and consequently enhance the antioxidant capacity of the cell, reduce oxidative damage, and maintain the functionality of the cell under conditions of drought stress. These mechanisms were also found to enhance the drought tolerance of other crop species, such as fenugreek.^61^ ^,^ ^74^

The present study showed that drought stress significantly increased the accumulation of MDA and H₂O₂. Similar findings have been reported in different crop plants^75^ ^,^ ^76^ Under water-deficit conditions, disturbances in cellular metabolic processes enhance the production of reactive oxygen species (ROS), resulting in H₂O₂ accumulation and membrane lipid peroxidation, as indicated by elevated MDA levels.^77^ In contrast, the exogenous application of *β*-carotene reduced the MDA and H₂O₂ contents in the present study. Comparable responses have been observed following *β*-carotene application in spinach,^76^ and spring wheat,^73^ suggesting its effectiveness in mitigating drought-induced oxidative damage. This protective effect may be attributed to the antioxidant activity of *β*-carotene, which enhances ROS detoxification, limits lipid peroxidation, and preserves membrane integrity and stability under stress conditions. The statistical results further indicated that drought stress and *β*-carotene application markedly affected the inorganic ion composition of fenugreek. Drought stress restricted nutrient uptake, particularly that of K⁺ and Ca^2^⁺, consistent with previous findings in mustard and camelina.^16^ The foliar application of growth regulator improved the drought tolerance by preserving ionic homeostasis, specifically by lowering Na^+^ accumulation and increasing K^+^ and Ca^2+^ uptake. This ionic regulation likely protects membrane integrity under water stress.^16^

In general, the drought tolerance conferred by *β*-carotene in fenugreek seems to be a result of synergistic ROS detoxification, antioxidant signaling, chloroplast protection and membrane stability. The integrated physiological and biochemical responses increase the ability of fenugreek plants to survive water shortfall and continue growth under drought stress.^61^

## 5. Conclusion

The present study demonstrated that drought stress markedly impaired the morphological, physiological, and ionic characteristics of fenugreek. Exposure to 50% field capacity increased H₂O₂ and MDA contents in both the Kasuri and Local varieties, indicating enhanced oxidative stress, lipid peroxidation, and cellular damage. However, foliar application of *β*-carotene effectively alleviated these adverse effects by improving plant growth, physiology, nutrient balance, and antioxidant defence. Among the tested treatments, 200  ppm *β*-carotene was the most effective in enhancing drought tolerance. Its application reduced MDA and H₂O₂ accumulation while increasing the activities of antioxidant enzymes, including superoxide dismutase, peroxidase, and catalase, as well as the level of ascorbic acid. These findings suggest that *β*-carotene can serve as a promising and sustainable approach for improving fenugreek performance under water-deficit conditions. Further research should investigate the molecular mechanisms underlying *β*-carotene-mediated drought tolerance, its interaction with other antioxidant pathways, and its long-term effects on yield and seed quality. Such information may support the development of climate-resilient cultivation strategies for fenugreek and other leguminous crops in arid and semi-arid regions.

## Data Availability

Data will be made available upon request.
